# Self-reported sleep patterns in a British population cohort^☆^

**DOI:** 10.1016/j.sleep.2013.10.015

**Published:** 2014-03

**Authors:** Yue Leng, Nick W.J. Wainwright, Francesco P. Cappuccio, Paul G. Surtees, Robert Luben, Nick Wareham, Carol Brayne, Kay-Tee Khaw

**Affiliations:** a Department of Public Health and Primary Care, Strangeways Research Laboratory, University of Cambridge, Cambridge, UK; b Division of Mental Health and Well-being, Warwick Medical School, University of Warwick, Coventry, UK; c Medical Research Council Epidemiology Unit, Institute of Metabolic Science, Cambridge, UK; d Department of Public Health and Primary Care, Institute of Public Health, University of Cambridge, Cambridge, UK

**Keywords:** Sleep quantity, Sleep patterns, Sleep research, England, Population, Epidemiology

## Abstract

**Objectives:**

Sleep patterns have been linked to various health outcomes, but sleep patterns in the British population have not been extensively reported. We aimed to describe the sleep characteristics reported by the European Prospective Investigation of Cancer (EPIC)-Norfolk participants, with a particular emphasis on the comparison of measures of sleep quantity.

**Methods:**

From 2006 to 2007, a total of 8480 participants aged 45–90 years reported sleep timing, nighttime sleep duration, and sleep difficulties. Time in bed (TIB) was calculated from the difference between rise time and bedtime, and sleep proportion was defined as the ratio of sleep duration and TIB.

**Results:**

On average, the reported TIB was more than 1.5 h longer than sleep durations. Compared to men, women spent 15 min longer in bed, but they slept for 11 min less and reported more sleep difficulties. In multivariate analysis sleep duration and TIB varied with socioeconomic factors, but sleep proportion was consistently lower among women, nonworkers, and older individuals, as well as those who were widowed, separated, or divorced; those who reported sleep difficulties and more frequently used sleep medication; and those who had lower education, poorer general health, or a major depressive disorder (MDD).

**Conclusions:**

Self-reported sleep duration and TIB have different meanings and implications for health. Sleep proportion may be a useful indicator of sleep patterns in the general population.

## Highlights

• Our study provides a subjective sleep profile of a large British population–based cohort.
• The reported time in bed (TIB) was more than 1.5 h longer than sleep duration.
• All sociodemographic factors varied with TIB and sleep duration.
• Sleep proportion may be a useful indicator of sleep patterns in this population.

## 1 Introduction

There are growing claims that we are now chronically sleep deprived due to increasing demands and pressures of modern society [1,2]. At the same time, recent findings from the Multinational Time Use Survey [3] suggested increased prevalence of long sleep duration among representative populations from 10 counties. Interestingly, a large Internet-based sleep survey covering 10,810 British adults suggested that few individuals opted for more sleep when given other alternatives, despite the reported sleep deficit [4]. Extensive epidemiologic evidence has associated both short and long self-reported sleep duration with a range of health outcomes including all-cause mortality [5,6], cardiovascular diseases [7], diabetes mellitus [8], hypertension [9], obesity [10], and impaired cognitive function [11]. Most of these studies have relied on a single question of sleep duration as the exposure, which led to concerns over the true meaning of this measure. For instance weekend sleep, usually known as the catch-up sleep, is longer than weekday sleep [12]. The time individuals spend in bed is influenced by many factors such as presleep lifestyles in addition to the sleep period itself, and time in bed (TIB) and sleep duration might have different implications for health. However, the two terms have been improperly used in many previous epidemiologic studies [13]. Understanding how to most adequately measure and characterize sleep patterns in the population may help clarify the link among sleep, well-being, and ill-health.

Few studies have provided a subjective sleep profile of the British population. One of the earlier studies [14] reported an average sleep duration of 7.6 h per 24 h in sleep diaries from 509 British adults. Another study using electroencephalogram records [15] found sleep durations of 7.3 h and 7.1 h among men and women, respectively. Both studies used objective measures of sleep, with the aim of presenting the age and sex distribution of the realistic sleep duration. A national survey [16] on perceived sleep in the British population took place in 2004 and covered a representative sample of 2000 adults. This survey addressed several sleep questions, including sleep duration, general sleep quality, and a range of sleep difficulties. Average sleep duration (7.04 h) was found to be 30 min less than the reported TIB. Descriptive studies from other countries have shown more variations. Most studies examined the sociodemographic distributions of sleep duration [17–19], though only a few studies presented the different aspects of sleep in detail [20–22].

Few studies have distinguished overall sleeping time between TIB and actual sleep durations [23] or weekday and weekend sleep time. Although the difference between TIB and sleep duration is obvious, the two terms have been easily confused with one another in previous studies with subjective sleep measures. More importantly, their potential different health implications indicate that it might be worth investigating the two sleep times separately in epidemiologic studies. Little is known about how each of these sleeping times vary by sociodemographic factors and how they may be linked to sleep quality. Our study aimed to provide a subjective profile of the sleep patterns in a British population, with a particular emphasis on the following questions. (1) How much do we sleep every night? (2) How is sleep duration different from the time we spend in bed? (3) How is weekend sleep different from weekday sleep? (4) How do these times vary by sociodemographic factors? (5) How does reported sleep quantity relate to sleep quality or sleep difficulties?

## 2 Methods

### 2.1 Study overview

**Fig. 1:**
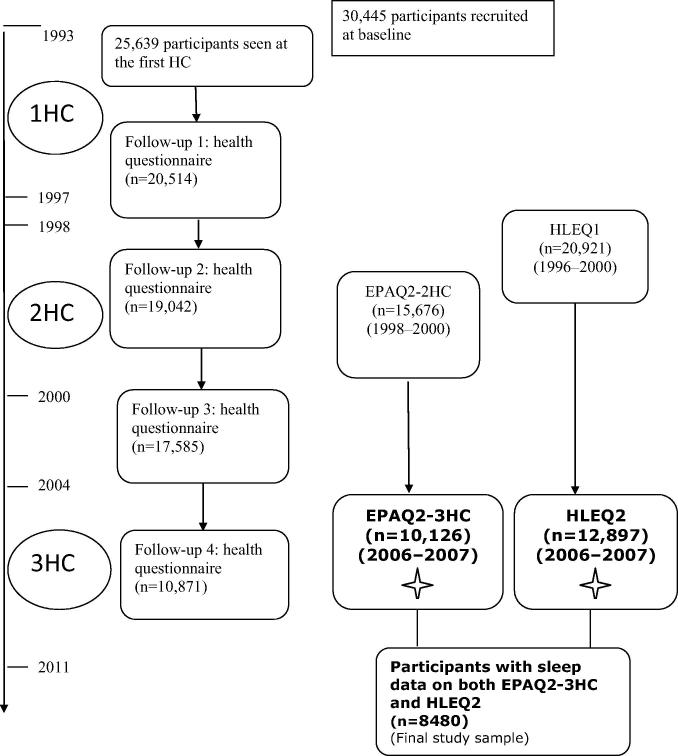
Overview of the European Prospective Investigation of Cancer (EPIC)-Norfolk study. *Abbreviations:* HC, health check; EPAQ2, EPIC Physical Activity Questionnaire; HLEQ2, Health and Lifestyle Questionnaire 2.  Questionnaires that contain measures on sleep described in our study.

Data were drawn from the European Prospective Investigation of Cancer (EPIC)-Norfolk prospective cohort study. The design and study methods of EPIC-Norfolk have been previously described [24,25]. Briefly 30,445 men and women aged 40–74 years were recruited into the EPIC-Norfolk study from 1993 to 1997 using general practice age-sex registers. There were 25,639 participants who attended the baseline health check. These participants were then followed up for two further health checks from 1996 to 2000 and 2006 to 2011. In between these health examinations, participants were sent questionnaires for completion and were expected to return them by mail (Fig. 1). The Norwich District Ethics Committee approved the study and all participants gave signed informed consent.

### 2.2 Measures of sleep

From 2006 to 2007, a total of 10,126 participants out of 13,969 eligible individuals completed the sleep questions in the EPIC physical activity questionnaire (EPAQ2-3HC). The questionnaire asked the following questions: “At what time do you normally get up?” “At what time do you normally go to bed?” The responses were separately obtained for an average weekday and weekend day over the previous year. For our study, 85 participants who reported unusual times of going to bed (6:00–18:00 pm) or getting up (12:00–24:00 pm/0:00–3:00 am) were omitted to make the interpretation more straightforward, leaving 10,041 individuals for analysis.

During the same period, some detailed questions on sleep patterns over the previous 4 weeks were asked in the Health and Lifestyle Questionnaire 2 (HLEQ2), completed by 12,897 participants. “Have you had difficulty getting to sleep at night?” “Have you woken-up during the night and had trouble getting back to sleep?” “Have you woken-up too early in the morning and had difficulty getting back to sleep?” “Have you taken any prescribed medicine to help with your sleep?” “On average, about how many hours have you slept each night?” Response categories for the first four questions included yes, usually/yes, sometimes/never, or rarely. Sleep duration was reported by hours and minutes.

### 2.3 Sociodemographic variables

#### 2.3.1 Baseline

The baseline questionnaire included the following sociodemographic variables: (1) social class (nonmanual/manual); (2) educational level (highest qualification attained: no qualifications, educated to the age of 16 years, educated to the age of 18 years, and educated to degree level; further collapsed into lower and higher); (3) presence of major depressive disorder (MDD) (yes/no) [26]; and (4) report of working night shift (yes/no).

#### 2.3.2 Follow-up three or four health questionnaires

Other sociodemographic variables included on third and fourth follow-up health questionnaires were: (1) working status (not working, working ⩽35 h/week, working >35 h/week); (2) marital status (single, married, other); (3) self-reported general health (excellent, very good, good, fair, poor); (4) self-reported preexisting health problems (yes/no; yes is any of the three: myocardial infarction, stroke, or cancer); and (5) coffee intake (⩽1 cup/day or >1 cup/day).

### 2.4 Analysis

Comparisons between TIB and sleep duration on their sociodemographic correlations and interrelationships with sleep quality were performed using data from 8480 participants who completed the sleep questions on both the EPAQ2-3HC and HLEQ2. TIB was derived from the differences between rise time and bedtime, and a weighted mean measure was calculated as 5/7*(TIB on a weekday)+2/7*(TIB on a weekend day). Sleep proportion was defined by the ratio of nighttime sleep duration and TIB. A sleep duration (or TIB) of longer than 14 h or less than 3 h was considered abnormal, and thereby was coded as missing values (*n* = 27). A sleep proportion of more than one also was disregarded (*n* = 319).

The sociodemographic associations of each sleep variable were examined by both univariate and multivariate analyses. Distributions of TIB, sleep duration, and sleep proportion were specifically picked up to clarify the overall sleep time. Interrelationships between measures of sleep quantity and sleep quality were then explored. The comparisons of normally distributed exposure variables were based on *t* tests for two groups and analysis of variance for three or more groups. The Wilcoxon rank sum test or the Kruskal–Wallis test was used for the ordinal variable (sleep proportion). Categorical variables were compared by Pearson product moment correlation *χ*^2^ tests. All statistical tests were two sided. Analyses were implemented in STATA version 12.0.

## 3 Results

### 3.1 Sleep timing: TIB

The average bedtime and rise time on weekdays was 22:41 pm and 7:17 am, respectively. On the weekend, individuals reported going to bed 12 min later (22:53 pm) but getting up 27 min later (7:44 am). This difference led to a TIB of 8 h and 36 min and 8 h and 51 min on weekdays and weekends, respectively. Supplementary Table 1 shows the distributions of the weekday and weekend bedtime and rise times. This Table also shows that older individuals spent longer in bed than younger individuals on weekdays, though the differences for weekends were smaller. Those who were working or worked for more hours spent much less TIB on weekdays; they also got up earlier but went to bed at similar times. However, these individuals tended to catch up on weekends by getting up more than 1 h later than they did on weekdays.

### 3.2 Sleep difficulties

Sleep difficulties more often were reported by women than men. There were 63.3% of who men reported never experiencing difficulty falling asleep vs 39.6% for women. Early awakening was the most common sleep complaint, reported by 652 (11.7%) men and 1076 (14.7%) women. Of the men and women, 9% and 14.7%, respectively, reported usually waking up during the night and having trouble going back to sleep. Supplementary Table 2 shows that younger individuals, working individuals, individuals with higher social class or higher education, and individuals with self-reported excellent general health reported fewer sleep problems and were less likely to take sleep medication.

### 3.3 TIB: sleep duration and sleep proportion

On average, the reported TIB was more than 1.5 h longer than sleep durations. Table 1 shows the different sociodemographic distributions of TIB, sleep duration, and sleep proportion. Women spent 15 min longer in bed but slept for 11 min less, and therefore had lower sleep proportion than men (79% vs 84%, respectively). Similarly older rather than younger individuals spent much more TIB (41 min and 26 min more for men and women, respectively), but they slept for less time (4 min and 18 min less for men and women, respectively) and had lower sleep proportion. Those who had lower education, poor general health, or MDD all showed the same patterns and had lower sleep proportion. The same results were revealed for those who were not working, who usually used sleep medication, as well as those who were widowed, separated, or divorced.

Multivariate analysis (Table 2) shows that age, sex, education, working status, marital status, general health, preexisting diseases, presence of MDD, and use of sleep medications were all independently associated with sleep proportion. Notably women or those with fair or poor health had much lower sleep proportion than the others, even after adjusting for all the covariates for women (*β* = −0.03 [95% confidence interval {CI} −0.04 to −0.03]) and those with poorer health (*β* = −0.04 [95% CI, −0.05 to −0.03]). Compared to those who used sleep medication, the nonusers had much higher sleep proportion (*β* = 0.07 [95% CI, 0.05–0.09]), but there was no significant difference between the frequency in which the medication was used.

### 3.4 Interrelationships between measures of sleep quantity and sleep quality

Table 3 summarizes the interrelationships between sleep quantity and sleep quality measures. All three reported sleep difficulties were associated with the reported sleep quantity and sleep proportion. Those who reported usually experiencing difficulty falling asleep, waking up during the night or early morning, and having difficulty going back to sleep had the lowest sleep proportion, with the highest being 0.70. Those who reported never experiencing these sleep problems had the highest sleep proportion, at approximately 0.87. These associations were unchanged after adjusting for the above sociodemographic factors.

## 4 Discussion

Our study provides a sleep profile of the EPIC-Norfolk population, a middle aged and older British population up to 75 years of age at baseline. The time individuals spent in bed every night was calculated from the reported bedtimes and rise times and on average was more than 1.5 h longer than sleep durations reported at a similar period. Compared to men, women had longer TIB, more sleep difficulties, and shorter sleep durations. Sleep duration and TIB varied with sociodemographic factors, but sleep proportion was consistently lower among women, nonworkers, and older individuals, as well as those who had lower education, poorer general health, and reported sleep difficulties.

To our knowledge, our study is the largest to describe sleep characteristics in a British population and the first to address the large gap between reported sleep duration and TIB. The EPIC-Norfolk participants are comparable to national population samples such as the population studied in the Health Survey of England 1993 in anthropometric variables, blood pressure, and serum lipids [24], as well as functional health [27]; they also both included participants with different socioeconomic statuses [28]. The wide range of sleep questions covered in validated questionnaires [29] allowed us to examine patterns of sleep in more detail to generate approaches for future studies.

There are several limitations to our study. First, our main analysis was based on 8480 participants who completed the EPAQ2 (3HC) and the HLEQ2. Compared to the other participants from the baseline sample (25,639 participants), these individuals were remarkably younger, more likely to be women, and of higher social and educational class. Although these differences should not affect the within-population associations of sociodemographic factors, the findings may not be generalizable to other populations. Second, there is likely to be measurement error from self-reported measures of TIB and sleep duration. Reported sleep may be a marker of general functional state or distress levels [30] and individuals who have health problems tend to report shorter sleep hours [31]. Besides we are comparing TIB, a computed variable, with sleep duration, which was determined by a single question. Therefore, the comparison was based on two different measurement approaches. Although our questionnaire has not been previously validated, the wording of our questions and the comparison approach were similar to those used in the Pittsburgh Sleep Quality Index, which has been extensively validated [32]. It also has been suggested that the use of times of going to bed, sleep onset, morning awakening, and rise times might help to clarify the confusing use of sleep duration and TIB [13,33]. From a practical standpoint, evaluation of sleep durations in the primary care setting relies on self-reported data from patients and reflects the individual’s own perception, which has validity in itself and has been associated with hard health end points [5,7,8]. Although objective measures of sleep may be feasible with new and available technologies, self-reported measure of sleep may still be a practical and useful measure in epidemiologic studies. Finally, our study presented results from descriptive analyses of a range of sociodemographic measures and differences in reported sleep patterns, which therefore require replication in other large-scale studies to provide better understanding of the relationships observed.

TIB was almost 2 h longer than sleep duration—more than that found in other studies using self-report measures [16,21,22]. Compared to a middle-aged Norwegian population and participants aged 55–101 years from the study of seven European countries, the EPIC-Norfolk participants reported longer TIB but similar sleep duration. This finding might be due to methodologic differences. The way we measured TIB was different from that in other studies. In addition, our study covered a sample aged 40–90 years, while the Hordaland Health Study [22] was conducted among individuals aged 40–45 years. However, our findings are in keeping with previous studies using objective measures of sleep [34,35]. Although our results need to be replicated by more population studies, they highlighted a potentially significant difference between TIB and sleep duration. The design of future epidemiologic studies should consider the inclusion of the assessment of TIB to avoid misinterpretation of sleeping time.

As expected, individuals spent much more TIB on weekends than on weekdays, especially among those who were younger or those who had a job. Individuals who were working spent more TIB on weekends by getting up more than 1 h later than they did on weekdays, reflective of the suggested weekend recovery sleep among workers [13]. The differences in TIB among sociodemographic groups seem to be greater on weekdays. It is possible that weekday sleep also may be a better predictor of future health risk if it is a more sensitive indicator of sociodemographic differences; further studies are needed to support this hypothesis.

**Fig. 2:**
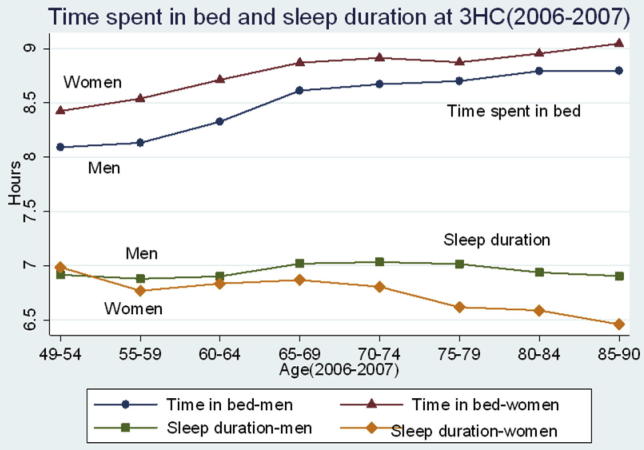
Relationship between time in bed and nighttime sleep durations by age at third health check (3HC) in 8480 men and women in Norwich, United Kingdom, from 2006 to 2007.

Our study suggested that women spent more TIB, went to bed earlier, and got up later than men. This finding is consistent with previous studies [21,22] which included self-reported sleep timing, as well as one study [36] including measures of actigraphy and sleep logs. Fig. 2 shows the separation of TIB and sleep duration by age and sex. Interestingly the sleep duration decreased in women and slightly increased in men with age; however, the TIB increased for both men and women. This finding is in line with a meta-analysis of objective studies covering 3500 individuals aged 5–102 years [37], which suggested a decreased total sleep time and sleep efficiency with increasing age. These results might help to explain the increased sleep duration among the elderly populations in previous studies using subjective sleep assessment [38,39,21], as older individuals may overestimate sleep durations due to long sleep latency [15]. Sleep efficiencies decreased with age, but the decrease among women was greater than that among men. In our study, women reported more sleep difficulties than men and it is possible that the reduction in sleep duration seen with aging was more of a concern among women.

Similarly the TIB and sleep durations of the participants varied with other sociodemographic factors; however, sleep proportion seemed to be consistently lower among women, nonworkers, and older individuals, as well as those who were widowed, separated, or divorced; those who reported sleep difficulties and more frequently used sleep medication; and those who had lower education, poorer general health, or an MDD. These associations were unchanged even after multivariate analysis, which included all the above factors. This finding suggests that the sociodemographic distributions of TIB and sleep durations should be separately explored in future observations. We are unaware of any previous studies of self-reported sleep that studied correlations of an individual’s sleep proportion. However, we have shown significantly decreased sleep proportion with increased sleep complaints among our participants, which suggested the potentially important role of sleep proportion as a relevant indicator of sleep in the population. It should be noted that those who took sleep medication slept for less time and had lower sleep proportion than those who never took such medication; however, the frequency of the medication did not make a notable difference. Although sleep medication is believed to improve sleep it is likely that the reported medication use was simply a reflection of their sleep problems, and thus the effects of sleep medication on sleep proportion are unclear.

## 5 Conclusion

Our study provides a sleep profile from a British population–based study. It highlights the large discrepancy between sleep duration and TIB and explores correlates of sleep proportion, which decreased with increasing age and was lower among women, nonworkers, and older individuals, as well as those who were widowed, separated, or divorced; those who reported sleep difficulties and more frequently used sleep medication; and those who had lower education, poorer general health, or an MDD. Those who reported frequent experience of sleep difficulties also had much lower sleep proportion. Our study addresses the increasing concerns over the meaning of sleep duration defined by one single question. Sleep quantity should be better defined and interpreted to aid in the understanding of its clinical implications in future studies. Sleep proportion might be an adequate additional indicator of overall sleep patterns.

## Funding sources

This work was supported by programme grants from the Medical Research Council UK G0401527 and Cancer Research UK (C864/A2883, C864/A8257). FPC leads the Sleep Health and Society Programme at the University of Warwick supported, in part, by the University of Warwick RDF and IAS. It has received funding by the NHS National Workforce Projects and the Economic and Social Research Council (ES/K002910/1).

## Conflict of interest

The ICMJE Uniform Disclosure Form for Potential Conflicts of Interest associated with this article can be viewed by clicking on the following link: http://dx.doi.org/10.1016/j.sleep.2013.10.015

Conflict of interest ICMJE Form for Disclosure of Potential Conflicts of Interest form.

## Figures and Tables

**Table 1:** Sociodemographic distributions of time in bed, sleep duration, and sleep proportion in Norwich, United Kingdom, from 2006 to 2007 (A, men; B, women).

	Time in bed	Sleep duration	Sleep proportion
(*A*)			
All (*N* = 3557)	8:32 (0.92)⁎⁎⁎	6:59 (1.11)⁎⁎⁎	0.84 (0.75–0.91)⁎⁎⁎

*Age* (*y*)			
49–54 (*n* = 131)	8:05 (0.76)⁎⁎⁎	6:55 (0.80)⁎⁎⁎	0.88 (0.81–0.92)⁎⁎⁎
55–64 (*n* = 1029)	8:16 (0.80)	6:54 (0.91)	0.86 (0.78–0.92)
65–74 (*n* = 1362)	8:38 (0.84)	7:02 (1.01)	0.83 (0.75–0.90)
75–84 (*n* = 934)	8:44 (0.95)	6:59 (1.16)	0.82 (0.73–0.90)
85–90 (*n* = 101)	8:48 (0.86)	6:54 (1.17)	0.79 (0.71–0.88)

*Social class*			
Nonmanual (*n* = 2293)	8:33 (0.89)	6:58 (1.01)	0.84 (0.75–0.91)
Manual (*n* = 1226)	8:32 (0.90)	6:57 (1.10)	0.84 (0.75–0.91)

*Education*			
Lower (*n* = 1149)	8:38 (0.97)⁎⁎⁎	6:56 (1.09)	0.83 (0.74–0.90)⁎⁎
Higher (*n* = 2406)	8:30 (0.85)	6:58 (1.01)	0.84 (0.76–0.91)

*Working status*			
Not working (*n* = 1565)	8:41 (0.88)⁎⁎⁎	7:00 (1.09)⁎⁎⁎	0.83 (0.74–0.90)⁎⁎⁎
⩽35 (*n* = 557)	8:30 (0.81)	7:04 (0.94)	0.85 (0.78–0.91)
>35 (*n* = 736)	8:04 (0.73)	6:47 (0.87)	0.86 (0.78–0.92)

*Marital status*			
Single (*n* = 81)	8:11 (0.82)⁎	6:53 (1.08)	0.86 (0.77–0.93)⁎
Married (*n* = 2242)	8:32 (0.86)	6:59 (1.02)	0.84 (0.75–0.91)
Others# (*n* = 268)	8:35 (0.96)	6:52 (1.13)	0.82 (0.72–0.90)

*General health*			
Good to excellent (*n* = 2147)	8:29 (0.84)⁎⁎⁎	7:01 (0.96)⁎⁎⁎	0.85 (0.76–0.91)⁎⁎⁎
Fair or poor (*n* = 450)	8:41 (0.99)	6:45 (1.26)	0.80 (0.69–0.89)

*Preexisting diseases*			
No (*n* = 2623)	8:30 (0.86)⁎⁎⁎	6:59 (0.99)⁎	0.84 (0.76–0.91)
Yes## (*n* = 558)	8:41 (0.92)	6:52 (1.11)	0.81 (0.71–0.89)

*MDD*			
No (*n* = 3103)	8:33 (0.88)	6:59 (1.01)⁎⁎⁎	0.84 (0.75–0.91)⁎⁎
Yes (*n* = 100)	8:38 (1.14)	6:37 (1.45)	0.79 (0.67–0.89)

*Shift work*			
No (*n* = 3509)	8:32 (0.89)	6:58 (1.04)	0.84 (0.75–0.91)
Yes (*n* = 48)	8:38 (0.99)	7:07 (0.93)	0.84 (0.78–0.91)

*Coffee intake*			
⩽1 cup/d (*n* = 1346)	8:35 (0.88)⁎	6:56 (1.07)	0.83 (0.74–0.90)⁎
>1 cup/d (*n* = 1764)	8:31 (0.88)	6:59 (0.99)	0.84 (0.76–0.91)

*Use of sleep medication*			
Usually (*n* = 115)	8:59 (1.09)⁎	6:31 (1.34)⁎⁎⁎	0.75 (0.62–0.87)⁎⁎⁎
Sometimes (*n* = 112)	8:41 (0.89)	6:17 (1.18)	0.72 (0.64–0.84)
Never (*n* = 3330)	8:31 (0.87)	7:01 (1.00)	0.84 (0.76–0.91)
			
(*B*)			
All (*N* = 4923)	8:47 (0.88)	6:48 (1.19)	0.79 (0.69–0.88)

*Age* (*y*)			
49–54 (*n* = 224)	8:26 (0.66)⁎⁎⁎	6:59 (1.02)⁎⁎⁎	0.85 (0.75–0.91)⁎⁎⁎
55–64 (*n* = 1734)	8:40 (0.80)	6:49 (1.09)	0.81 (0.72–0.89)
65–74 (*n* = 1782)	8:53 (0.82)	6:50 (1.11)	0.79 (0.69–0.87)
75–84 (*n* = 1063)	8:54 (0.94)	6:37 (1.19)	0.76 (0.66–0.85)
85–90 (*n* = 120)	9:03 (0.93)	6:28 (1.42)	0.72 (0.60–0.84)

*Social class*			
Nonmanual (*n* = 3206)	8:47 (0.84)	6:47 (1.12)⁎	0.79 (0.69–0.88)⁎
Manual (*n* = 1660)	8:49 (0.88)	6:43 (1.16)	0.78 (0.68–0.87)

*Education*			
Lower (*n* = 2232)	8:52 (0.86)⁎⁎⁎	6:41 (1.18)⁎⁎⁎	0.77 (0.67–0.86)⁎⁎⁎
Higher (*n* = 2691)	8:45 (0.85)	6:49 (1.09)	0.80 (0.71–0.88)

*Working status*			
Not working (*n* = 2345)	8:53 (0.83)⁎⁎⁎	6:46 (1.16)⁎⁎⁎	0.78 (0.68–0.86)⁎⁎⁎
⩽35 (*n* = 1119)	8:36 (0.79)	6:50 (1.08)	0.82 (0.72–0.89)
>35 (*n* = 360)	8:16 (0.70)	6:43 (0.95)	0.83 (0.75–0.90)

*Marital status*			
Single (*n* = 156)	8:43 (0.86)⁎⁎⁎	6:43 (1.05)⁎⁎	0.78 (0.71–0.86)⁎⁎⁎
Married (*n* = 2482)	8:46 (0.80)	6:51 (1.08)	0.80 (0.71–0.88)
Others (*n* = 891)	8:46 (0.90)	6:37 (1.20)	0.77 (0.67–0.86)

*General health*			
Good to excellent (*n* = 2980)	8:44 (0.80)⁎⁎⁎	6:50 (1.06)⁎⁎⁎	0.80 (0.71–0.88)⁎⁎⁎
Fair or poor (*n* = 553)	8:55 (0.96)	6:29 (1.32)	0.74 (0.64–0.84)

*Preexisting diseases*			
No (*n* = 3561)	8:45 (0.83)⁎⁎⁎	6:48 (1.11)	0.80 (0.70–0.88)⁎⁎⁎
Yes (*n* = 778)	8:53 (0.90)	6:43 (1.15)	0.78 (0.67–0.86)

*MDD*			
No (*n* = 4184)	8:47 (0.84)⁎⁎⁎	6:46 (1.12)	0.79 (0.69–0.88)⁎
Yes (*n* = 273)	8:59 (0.99)	6:43 (1.25)	0.76 (0.66–0.86)

*Shift work*			
No (*n* = 4887)	8:47 (0.85)	6:46 (1.13)	0.79 (0.69–0.88)
Yes (*n* = 36)	8:52 (1.14)	6:47 (1.30)	0.77 (0.69–0.87)

*Coffee intake*			
⩽1 cup/d (*n* = 2053)	8:49 (0.86)	6:45 (1.13)	0.79 (0.68–0.87)⁎
>1 cup/d (*n* = 2317)	8:46 (0.84)	6:48 (1.11)	0.80 (0.70–0.87)

*Use of sleep medication*			
Usually (*n* = 243)	9:08 (0.91)⁎	6:23 (1.38)⁎⁎⁎	0.71 (0.61–0.81)⁎⁎⁎
Sometimes (*n* = 324)	9:04 (0.91)	6:21 (1.24)	0.71 (0.61–0.81)
Never (*n* = 4356)	8:46 (0.83)	6:50 (1.09)	0.80 (0.71–0.88)

*Abbreviations:* y, years; MDD, major depressive disorder; d, day. For all, ^∗^ is for comparisons between men and women. Comparisons of time in bed and sleep duration were based on *t* tests or one-way analysis of variance, and comparisons of sleep proportion were based on Wilcoxon rank sum test or Kruskal–Wallis test. All the *P* values show the overall comparisons among different sociodemographic groups. Time in bed and sleep duration are presented as mean (hour: minutes) (standard deviation [hour]); sleep proportion is presented as median (interquartile range). Sleep proportion = night time sleep duration/time in bed. All the variables were concurrently measured from 2006 to 2007, except for social class, education, MDD, and shift work, which were defined at baseline.

# Widowed, separated, or divorced.

## Self-reported myocardial infarction, stroke, or cancer.

⁎ *P* < .05.

⁎⁎ *P* < .01.

⁎⁎⁎ *P* < .001.

**Table 2:** Multivariate regressions for time in bed, sleep duration, and sleep proportion in Norwich, United Kingdom, from 2006 to 2007.

	Time in bed (min)	*P* value	Sleep duration (min)	*P* value	Sleep proportion	*P* value
Age (per 10-year increase)	1.2 (−1.2 to 3.6)	.251	−5.4 (−8.4 to −2.4)⁎⁎	<.001	−0.01 (−0.02 to −0.01)⁎⁎⁎	<.001

*Sex*		<.001		<.001		<.001
Men	Reference					
Women	9.0 (6.0–12.0)⁎⁎⁎		−9.6 (−13.8 to −6.0)⁎⁎⁎		−0.03 (−0.04 to −0.03)⁎⁎⁎	

*Social class*		.057		.064		.189
Nonmanual	Reference					
Manual	−1.8 (−4.8 to 1.2)		−3.6 (−7.8 to 0.0)		0 (−0.01 to 0.00)	

*Education*		.022		.010		<.001
Lower	Reference					
Higher	−3.6 (−6.6 to −0.6)⁎		4.8 (1.2–9.0)⁎⁎		0.01 (0.01–0.02)⁎⁎⁎	

*Working status*		<.001		<.001		<.001
Not working	Reference					
Working ⩽35 h/w	−15.0 (−18.6 to −10.8)⁎⁎⁎		−4.8 (−9.6 to 0.0)		0.01 (0.00–0.02)⁎⁎	
Working >35 h/w	−32.4 (−37.8 to −27.6)⁎⁎⁎		−20.4 (−27.0 to −13.8)⁎⁎⁎		0.01 (0.00–0.02)⁎	

*Marital status*		.004		<.001		.050
Single	Reference					
Married	10.2 (3.0–18.0)⁎⁎		6.6 (−3.0 to 15.6)		0.00 (−0.02 to 0.01)	
Others#	6.0 (−1.8 to 14.4)		−3.6 (−13.8 to 6.6)		−0.01 (−0.03 to 0.00)	

*General health*		.017		<.001		<.001
Good to excellent	Reference					
Fair or poor	4.8 (0.6–9.0)⁎		−15.0 (−19.8 to −9.6)⁎⁎⁎		−0.04 (−0.05 to −0.03)⁎⁎⁎	

*Preexisting diseases*		.035		.130		.008
No	Reference					
Yes##	4.2 (0.0–7.8)⁎		−3.6 (−8.4 to 1.2)		−0.01 (−0.02 to −0.00)⁎⁎	

*MDD*		.018		.075		.001
No	Reference					
Yes	8.4 (1.2–15.0)⁎		−7.8 (−16.8 to 0.6)		−0.03 (−0.04 to −0.01)⁎⁎⁎	

*Shift work*		.800		.077		.088
No	Reference					
Yes	1.8 (−11.4 to 15.0)		15.0 (−1.8 to 32.4)		0.03 (−0.00 to 0.06)	

*Coffee intake*		.347		.883		.660
⩽1 cup/d	Reference					
>1 cup/d	−1.2 (−4.2 to 1.2)		0.0 (−3.6 to 3.6)		0.00 (−0.01 to 0.01)	

*Use of sleep medication*		<.001		<.001		<.001
Usually	Reference					
Sometimes	−1.8 (−12.0 to 8.4)		1.8 (−10.8 to 15.0)		0.00 (−0.02 to 0.03)	
Never	−15.6 (−23.4 to −7.8)⁎⁎⁎		25.2 (15.0–35.4)⁎⁎⁎		0.07 (0.05–0.09)	

*Abbreviations:* min, minutes; h, hours; w, week; MDD, major depressive disorder; d, day. Results are presented as *β* (confidence interval) in minutes. Sample sizes: time in bed *N* = 5055, sleep duration *N* = 5842, sleep proportion *N* = 4866. Sleep proportion = nighttime sleep duration/time in bed. Each linear regression model includes all the above factors. All variables were concurrently measured from 2006 to 2007, except for social class, education, MDD and shift work, which were defined at baseline.

⁎ *P* < .05.

⁎⁎ *P* < .01.

⁎⁎⁎ *P* < .001.

# Widowed, separated, or divorced.

## Self-reported myocardial infarction, stroke, or cancer.

**Table 3:** Interrelationships between measures of sleep quality and sleep quantity in Norwich, United Kingdom, from 2006 to 2007.

	Time in bed	Sleep duration	Sleep proportion		Time in bed	Sleep duration	Sleep proportion
	Mean (SD) h	Mean (SD) h	Median (IQR)		Mean (SD) h	Mean (SD) h	Median (IQR)
*Difficulty falling asleep*				*Difficulty falling asleep*			
Usually (*n* = 179)	8:40 (1.20)⁎⁎⁎	5:46 (1.35)⁎⁎⁎	0.65 (0.56–0.80)⁎⁎⁎	Usually (*n* = 478)	8:57 (1.05)⁎⁎⁎	5:29 (1.16)⁎⁎⁎	0.63 (0.53–0.71)⁎⁎⁎
Sometimes (*n* = 1104)	8:36 (0.86)	6:41 (1.00)	0.79 (0.71–0.87)	Sometimes (*n* = 2494)	8:49 (0.82)	6:40 (1.04)	0.77 (0.68–0.84)
Never (*n* = 2274)	8:30 (0.86)	7:12 (0.90)	0.87 (0.80–0.92)	Never (*n* = 1951)	8:43 (0.82)	7:14 (0.94)	0.84 (0.77–0.91)

*Waking up during the night*				*Waking up during the night*			
Usually (*n* = 298)	8:39 (1.03)⁎⁎⁎	5:46 (1.13)⁎⁎⁎	0.68 (0.58–0.76)⁎⁎⁎	Usually (*n* = 690)	8:52 (0.97)⁎⁎⁎	5:34 (1.12)⁎⁎⁎	0.63 (0.55–0.73)⁎⁎⁎
Sometimes (*n* = 1933)	8:36 (0.85)	6:55 (0.96)	0.82 (0.74–0.89)	Sometimes (*n* = 2993)	8:49 (0.81)	6:47 (0.99)	0.78 (0.70–0.85)
Never (*n* = 1326)	8:26 (0.88)	7:19 (0.86)	0.88 (0.82–0.93)	Never (*n* = 1240)	8:42 (0.86)	7:26 (0.86)	0.87 (0.80–0.93)

*Early awakening*				*Early awakening*			
Usually (*n* = 397)	8:28 (1.02)⁎⁎⁎	5:51 (1.11)⁎⁎⁎	0.70 (0.60–0.79)⁎⁎⁎	Usually (*n* = 674)	8:46 (0.99)⁎⁎⁎	5:35 (1.11)⁎⁎⁎	0.64 (0.56–0.75)⁎⁎⁎
Sometimes (*n* = 1878)	8:34 (0.85)	6:55 (0.91)	0.82 (0.74–0.89)	Sometimes (*n* = 2738)	8:49 (0.81)	6:41 (0.97)	0.77 (0.69–0.84)
Never (*n* = 1282)	8:32 (0.89)	7:24 (0.87)	0.88 (0.83–0.94)	Never (*n* = 1511)	8:45 (0.84)	7:27 (0.88)	0.87 (0.80–0.93)

*Abbreviations:* SD, standard deviation; h, hours; IQR, interquartile range. Comparisons of time in bed and sleep duration were based on one-way analysis of variance, and comparisons of sleep proportion were based on Kruskal–Wallis test. All the *P* values show the overall comparisons among different levels of sleep difficulties. Time in bed and sleep duration are presented as mean (h:min) (SD [h]) and sleep proportion is presented as median (IQR). Sleep proportion = nighttime sleep duration/time in bed. ^∗^*P* < .05. ^∗∗^*P* < .01.

⁎⁎⁎ *P* < .001.

## Appendix A Supplementary data

Supplementary data 2 These document contains Supplementary Tables 1 and 2.

## References

[1] Bonnet M.H., Arand D.L. (1995). We are chronically sleep deprived. Sleep.

[2] Banks S., Dinges D.F. (2007). Behavioral and physiological consequences of sleep restriction. J Clin Sleep Med.

[3] Bin Y.S., Marshall N.S., Glozier N. (2013). Sleeping at the limits: the changing prevalence of short and long sleep durations in 10 countries. Am J Epidemiol.

[4] Anderson C., Horne J.A. (2008). Do we really want more sleep? A population-based study evaluating the strength of desire for more sleep. Sleep Med.

[5] Grandner M.A., Hale L., Moore M., Patel N.P. (2010). Mortality associated with short sleep duration: the evidence, the possible mechanisms, and the future. Sleep Med Rev.

[6] Cappuccio F.P., D’Elia L., Strazzullo P., Miller M.A. (2010). Sleep duration and all-cause mortality: a systematic review and meta-analysis of prospective studies. Sleep.

[7] Amagai Y., Ishikawa S., Gotoh T., Kayaba K., Nakamura Y., Kajii E. (2010). Sleep duration and incidence of cardiovascular events in a Japanese population: the Jichi Medical School Cohort Study. J Epidemiol.

[8] Gangwisch J.E., Heymsfield S.B., Boden-Albala B., Buijs R.M., Kreier F., Pickering T.G. (2007). Sleep duration as a risk factor for diabetes incidence in a large US sample. Sleep.

[9] Gottlieb D.J., Redline S., Nieto J., Baldwin C.M., Newman A.B., Resnick H.E. (2006). Association of usual sleep duration with hypertension: the sleep heart health study. Sleep.

[10] Patel S.R., Hu F.B. (2008). Short sleep duration and weight gain: a systematic review. Obesity.

[11] Ferrie J.E., Shipley M.J., Akbaraly T.N., Marmot M.G., Kivimäki M., Singh-Manoux A. (2011). Change in sleep duration and cognitive function: findings from the Whitehall II Study. Sleep.

[12] Hale L. (2005). Who has time to sleep?. J Public Health (Oxf).

[13] Habitual Horne J. (2010). “Short sleep”: six hours is “safe”. J Sleep Res.

[14] Tune G.S. (1969). Sleep and wakefulness in 509 normal human adults. Br J Med Psychol.

[15] Hume K.I., Van F., Watson A. (1998). A field study of age and gender differences in habitual adult sleep. J Sleep Res.

[16] Groeger J.A., Zijlstra F.R.H., Dijk D.J. (2004). Sleep quantity, sleep difficulties and their perceived consequences in a representative sample of some 2000 British adults. J Sleep Res.

[17] Ohida T., Kamal A., Uchiyama M., Kim K., Takemura S., Sone T. (2001). The influence of lifestyle and health status factors on sleep loss among the Japanese general population. Sleep.

[18] Kronholm E., Härmä M., Hublin C., Aro A.R., Partonen T. (2006). Self-reported sleep duration in Finnish general population. J Sleep Res.

[19] Krueger P.M., Friedman E.M. (2009). Sleep duration in the United States: a cross-sectional population-based study. Am J Epidemiol.

[20] National Sleep Foundation. 2003 Sleep in America Poll: Sleep and aging. Washington (DC): The Foundation; 2003 Mar. Available from: http://www.sleepfoundation.org/article/sleep-america-polls/2003-sleep-and-aging.

[21] Ohayon M.M. (2004). Interactions between sleep normative data and sociocultural characteristics in the elderly. J Psychosom Res.

[22] Ursin R., Bjorvatn B., Holsten F. (2005). Sleep duration, subjective sleep need, and sleep habits of 40- to 45-year-olds in the Hordaland Health Study. Sleep.

[23] Ferrie J.E., Kumari M., Salo P., Singh-Manoux A., Kivimäki M. (2011). Sleep epidemiology—a rapidly growing field. Int J Epidemiol.

[24] Day N., Oakes S.A., Luben R., Khaw K.-T., Bingham S., Welch A. (1999). EPIC-Norfolk: study design and characteristics of the cohort. Br J Cancer.

[25] Hayat S., Luben R., Keevil V., Moore S., Dalzell N., Bhaniani A. (2013). Cohort profile: a prospective cohort study of objective physical and cognitive capability and visual health in an ageing population of men and women in Norfolk (EPIC-Norfolk 3). Int J Epidemiol.

[26] Surtees P.G., Wainwright N.W., Luben R., Wareham N.J., Bingham S.A., Khaw K.-T. (2008). Depression and ischemic heart disease mortality: evidence from the EPIC-Norfolk United Kingdom prospective cohort study. Am J Psychiatry.

[27] Surtees P.G., Wainwright N.W., Khaw K.T. (2004). Obesity, confidant support and functional health: cross-sectional evidence from the EPIC-Norfolk cohort. Int J Obes Relat Metab Disord.

[28] Wainwright N.W., Surtees P.G. (2004). Places, people, and their physical and mental functional health. J Epidemiol Commun Health.

[29] Wareham N.J., Jakes R.W., Rennie K.L., Schuit J., Mitchell J., Hennings S. (2002). Validity and repeatability of the EPIC-Norfolk physical activity questionnaire. Int J Epidemiol.

[30] Lavie P. (2009). Self-reported sleep duration—what does it mean?. J Sleep Res.

[31] Lauderdale D. (2008). Sleep duration: how well do self-reports reflect objective measures? The CARDIA Sleep Study. Epidemiology.

[32] Buysse D.J., Reynolds C.F., Monk T.H., Berman S.R., Kupfer D.J. (1989). The Pittsburgh Sleep Quality Index: a new instrument for psychiatric practice and research. Psychiatr Res.

[33] Horne J. (2011). The end of sleep: “sleep debt” versus biological adaptation of human sleep to waking needs. Bio Psychol.

[34] Lauderdale D.S., Knutson K.L., Yan L.L., Rathouz P.J., Hulley S.B., Sidney S. (2006). Objectively measured sleep characteristics among early-middle-aged adults: the CARDIA study. Am J Epidemiol.

[35] Van Den Berg J.F., Van Rooij F.J.A., Vos H., Tulen J.H.M., Hofman A., Miedema H.M.E. (2008). Disagreement between subjective and actigraphic measures of sleep duration in a population-based study of elderly persons. J Sleep Res.

[36] Reyner L.A., Horne J.A., Reyner A. (1995). Gender- and age-related differences in sleep determined by home-recorded sleep logs and actimetry from 400 adults. Sleep.

[37] Ohayon M.M., Carskadon M.A., Guilleminault C., Vitiello M.V. (2004). Meta-analysis of quantitative sleep parameters from childhood to old age in healthy individuals: developing normative sleep values across the human lifespan. Sleep.

[38] Kripke D.F., Simons R.N., Garfinkel L., Hammond E.C. (1979). Short and long sleep and sleeping pills. Is increased mortality associated?. Arch Gen Psychiatry.

[39] Tamakoshi A., Ohno Y. (2004). Self-reported sleep duration as a predictor of all-cause mortality: results from the JACC study, Japan. Sleep.

