# Supplementary material for: Self-reported sleep patterns in a British population cohort
Source: Sleep Med. 2014 Mar;15(3):295–302. doi: 10.1016/j.sleep.2013.10.015 (PMC3988958; doi:10.1016/j.sleep.2013.10.015)
Supplement: Conflict of interest — ICMJE Form for Disclosure of Potential Conflicts of Interest form. [file mmc1.zip › FC_COI form.pdf]

The full content of this file cannot be displayed with your current PDF viewer. Please update to the latest possible version to view this document.
