# Supplementary material for: Self-reported sleep patterns in a British population cohort
Source: Sleep Med. 2014 Mar;15(3):295–302. doi: 10.1016/j.sleep.2013.10.015 (PMC3988958; doi:10.1016/j.sleep.2013.10.015)
Supplement: Supplementary data 2 — These document contains Supplementary Tables 1 and 2. [file mmc2.docx]

|  | Bedtime |  | Rise time |  | Time in bed |  |
| --- | --- | --- | --- | --- | --- | --- |
|  | Weekday | Weekend | Weekday | Weekend | Weekday | Weekend |
| **All (n=4271)** | 22:46 (0.82)*** | 22:57 (0.85)*** | 7:13 (0.91)*** | 7:40 (0.88)*** | 8:27 (0.97)*** | 8:43 (0.97)*** |
| **Age (y)** |  |  |  |  |  |  |
| 45–44 (n=157) | 22:57 (0.77) | 23:21 (0.91) | 6:46 (0.82)*** | 7:53 (0.94)*** | 7:49 (0.81)*** | 8:34 (1.03)*** |
| 55–64 (n=1209) | 22:46 (0.81) | 23:07 (0.81) | 6:53 (1.01) | 7:46 (0.94) | 8:05 (0.91) | 8:38 (0.94) |
| 65–74 (n=1531) | 22:48 (0.81) | 22:56 (0.84) | 7:23 (0.81) | 7:39 (0.83) | 8:36 (0.89) | 8:43 (0.92) |
| 75–90 (n=1175) | 22:41 (0.83) | 22:46 (0.86) | 7:25 (0.83) | 7:34 (0.86) | 8:44 (1.01) | 8:48 (1.07) |
| **Social class** | |  |  |  |  |  |
| Nonmanual (n=2698) | 22:48 (0.82)*** | 22:58 (0.84) | 7:16 (0.86)*** | 7:41 (0.84) | 8:28 (0.95) | 8:43 (0.95) |
| Manual (n=1528) | 22:41 (0.82) | 22:56 (0.88) | 7:08 (1.00) | 7:39 (0.95) | 8:26 (1.01) | 8:43 (1.02) |
| **Education** |  |  |  |  |  |  |
| Lower (n=1426) | 22:39 (0.86)*** | 22:50 (0.90)*** | 7:14 (0.99) | 7:38 (0.93)* | 8:34 (1.05)*** | 8:47 (1.06)* |
| Higher (n=2843) | 22:49 (0.79) | 23:00 (0.82) | 7:13 (0.87) | 7:41 (0.85) | 8:24 (0.92) | 8:41 (0.92) |
| **Occupation** | |  |  |  |  |  |
| Working (n=808) | 22:46 (0.81) | 23:06 (0.82)*** | 6:44 (0.91)*** | 7:44 (0.92)* | 7:58 (0.83)*** | 8:38 (0.94)* |
| Not working (n=2114) | 22:47 (0.81) | 22:55 (0.85) | 7:23 (0.83) | 7:39 (0.85) | 8:35 (0.92) | 8:44 (0.97) |
| **Marital status** | |  |  |  |  |  |
| Single (n=94) | 22:54 (1.20)*** | 23:07 (1.23)*** | 7:07 (1.33)*** | 7:24 (1.28)*** | 8:13 (0.90) | 8:19 (0.86) |
| Married (n=2515) | 22:46 (0.78) | 22:58 (0.82) | 7:12 (0.89) | 7:41 (0.85) | 8:25 (0.94) | 8:43 (0.95) |
| Others# (n=301) | 22:51 (0.83) | 22:58 (0.90) | 7:21 (0.81) | 7:38 (0.83) | 8:30 (0.97) | 8:40 (0.99) |
| **Weekly working hours** | |  |  |  |  |  |
| ≦35 h (n=630) | 22:44 (0.75) | 22:59 (0.83)** | 7:04 (0.84)*** | 7:40 (0.92) | 8:20 (0.87)*** | 8:40 (0.89) |
| >35 h (n=882) | 22:45 (0.77) | 23:08 (0.74) | 6:36 (0.92) | 7:46 (0.91) | 7:50 (0.81) | 8:37 (0.92) |
| **General health** | |  |  |  |  |  |
| Excellent (n=200) | 22:53 (0.85)*** | 23:05 (0.87)*** | 7:06 (0.93) | 7:39 (0.86) | 8:11 (0.86)*** | 8:34 (0.95)*** |
| Very good (n=980) | 22:49 (0.76) | 23:01 (0.78) | 7:09 (0.89) | 7:39 (0.86) | 8:20 (0.88) | 8:39 (0.92) |
| Good (n=1223) | 22:45 (0.81) | 22:56 (0.84) | 7:13 (0.91) | 7:40 (0.86) | 8:27 (0.95) | 8:44 (0.95) |
| Fair (n=469) | 22:45 (0.83) | 22:55 (0.91) | 7:21 (0.87) | 7:46 (0.91) | 8:35 (0.97) | 8:50 (0.99) |
| Poor (n=48) | 22:39 (1.22) | 22:56 (1.30) | 7:17 (0.97) | 7:33 (0.94) | 8:37 (1.48) | 8:36 (1.45) |
| **Self-reported preexisting diseases** | | |  |  |  |  |
| No (n=3076) | 22:52 (0.78) | 22:58 (0.83)** | 7:02 (0.92)*** | 7:40 (0.88) | 8:24 (0.95)*** | 8:41 (0.94)* |
| Yes## (n=672) | 22:50 (0.78) | 22:52 (0.88) | 7:15 (0.86) | 7:38 (0.88) | 8:38 (0.99) | 8:47 (1.02) |

Supplementary Table 1 Sleep characteristics measured in the European Prospective Investigation of Cancer (EPIC) Physical Activity Questionnaire 2 on health check 3 (men).

Abbreviation: y, years.

Total number of men and women was 10,126. There were 85 participants who reported unusual times of going to bed (6:00–18:00) or getting up (12:00–24:00/0:00–3:00) and were omitted to make the interpretation more straightforward, leaving 10,041 individuals for analysis (men 4271; women 5770).

All results are presented as mean (hour:minutes) (standard deviation [hour]).

**P*<.05; ***P*<.005; ****P*<.001.

For all, *is for comparisons between men and women. Comparisons were based on *t* tests or 1-way analysis of variance.

#Widowed, separated, or divorced.

##Self-reported myocardial infarction, stroke, or cancer.

Supplementary Table 1 Sleep characteristics measured in the European Prospective Investigation of Cancer (EPIC) Physical Activity Questionnaire 2 on health check 3 (women).

|  | Bedtime |  | Rise time |  | Time in bed |  |
| --- | --- | --- | --- | --- | --- | --- |
|  | Weekday | Weekend | Weekday | Weekend | Weekday | Weekend |
| **All (n=5770)** | 22:37 (0.77) | 22:49 (0.80) | 7:20 (0.82) | 7:46 (0.84) | 8:43 (0.92) | 8:57 (0.92) |
| **Age (y)** |  |  |  |  |  |  |
| 45–44 (n=269) | 22:40 (0.73)*** | 23:07 (0.78)*** | 6:50 (0.74)*** | 8:04 (0.90)* | 8:11 (0.89)*** | 8:57 (0.98)*** |
| 55–64 (n=1945) | 22:40 (0.72) | 22:58 (0.77) | 7:13 (0.87) | 7:55 (0.84) | 8:34 (0.90) | 8:57 (0.89) |
| 65–74 (n=1930) | 22:38 (0.74) | 22:46 (0.75) | 7:28 (0.87) | 7:42 (0.81) | 8:50 (0.84) | 8:57 (0.89) |
| 75–90 (n=1321) | 22:31 (0.84) | 22:35 (0.84) | 7:27 (0.77) | 7:36 (0.81) | 8:55 (0.98) | 9:06 (1.01) |
| **Social class** | |  |  |  |  |  |
| Nonmanual (n=3710) | 22:40 (0.75)*** | 22:50 (0.78)* | 7:23 (0.78)*** | 7:48 (0.83)* | 8:43 (0.91) | 8:58 (0.91) |
| Manual (n=1975) | 22:32 (0.79) | 22:47 (0.84) | 7:16 (0.87) | 7:44 (0.85) | 8:44 (0.95) | 8:57 (0.95) |
| **Education** | |  |  |  |  |  |
| Lower (n=2703) | 22:33 (0.78)*** | 22:45 (0.82)*** | 7:20 (0.85) | 7:45 (0.85)* | 8:48 (0.95)*** | 8:59 (0.95)* |
| Higher (n=3064) | 22:41 (0.75) | 22:52 (0.78) | 7:20 (0.79) | 7:48 (0.83) | 8:40 (0.90) | 8:56 (0.90) |
| **Occupation** | |  |  |  |  |  |
| Working (n=847) | 22:38 (0.74) | 23:01 (0.77)*** | 6:53 (0.76)*** | 7:55 (0.84)*** | 8:14 (0.85)*** | 8:54 (0.89) |
| Not working (n=3116) | 22:39 (0.76) | 22:49 (0.79) | 7:26 (0.77) | 7:45 (0.82) | 8:48 (0.88) | 8:57 (0.91) |
| **Marital status** | |  |  |  |  |  |
| Single (n=180) | 22:40 (0.80)*** | 22:49 (0.83)*** | 7:16 (0.88) | 7:42 (0.98)*** | 8:36 (0.88)*** | 8:52 (0.97)*** |
| Married (n=2760) | 22:40 (0.70) | 22:53 (0.74) | 7:20 (0.80) | 7:50 (0.79) | 8:40 (0.87) | 8:57 (0.86) |
| Others# (n=1010) | 22:37 (0.85) | 22:46 (0.88) | 7:18 (0.81) | 7:39 (0.89) | 8:42 (0.98) | 8:52 (0.99) |
| **Weekly working hours** | |  |  |  |  |  |
| ≦35h (n=1274) | 22:40 (0.73) | 23:00 (0.74) | 7:03 (0.76)*** | 7:55 (0.81) | 8:23 (0.80)*** | 8:55 (0.84) |
| >35h (n=415) | 22:38 (0.71) | 23:02 (0.79) | 6:38 (0.76) | 7:54 (0.90) | 8:00 (0.84) | 8:52 (0.94) |
| **General health** | |  |  |  |  |  |
| Excellent (n=288) | 22:41 (0.75)*** | 22:58 (0.78)*** | 7:09 (0.78)* | 7:49 (0.81) | 8:28 (0.95)*** | 8:51 (0.94)*** |
| Very good (n=1382) | 22:40 (0.69) | 22:53 (0.73) | 7:15 (0.78) | 7:47 (0.81) | 8:36 (0.83) | 8:54 (0.84) |
| Good (n=1639) | 22:38 (0.74) | 22:50 (0.77) | 7:20 (0.79) | 7:45 (0.83) | 8:42 (0.87) | 8:55 (0.89) |
| Fair (n=573) | 22:37 (0.86) | 22:48 (0.85) | 7:28 (0.86) | 7:49 (0.86) | 8:51 (1.03) | 9:01 (0.99) |
| Poor (n=70) | 22:38 (1.12) | 22:45 (1.32) | 7:44 (0.88) | 8:04 (0.96) | 9:05 (1.09) | 9:13 (1.28) |
| **Self-reported preexisting diseases** | | |  |  |  |  |
| No (n=4081) | 22:44 (0.72)* | 22:51 (0.77)** | 7:13 (0.80)*** | 7:47 (0.83) | 8:40 (0.90)*** | 8:56 (0.89)* |
| Yes## (n=906) | 22:41 (0.77) | 22:46 (0.83) | 7:20 (0.79) | 7:47 (0.85) | 8:49 (0.95) | 9:06 (0.98) |

Abbreviation: y, years.

Total number of men and women was 10,126. There were 85 participants who reported unusual times of going to bed (6:00–18:00) or getting up (12:00–24:00/0:00–3:00) and were omitted to make the interpretation more straightforward, leaving 10,041 individuals for analysis (men 4271; women 5770).

Bedtime and rise time: results are presented as mean (hour:minutes) (standard deviation [hour]).

Time in bed: results are presented as mean (hour) (standard deviation [hour]).

**P*<.05; ***P*<.005; ****P*<.001.

For all, *is for comparisons between men and women. Comparisons were based on *t* tests or 1-way analysis of variance.

#Widowed, separated, or divorced.

## Self-reported myocardial infarction, stroke, or cancer

| Supplementary Table 2 Sleep characteristics measured in the Health and Lifestyle Questionnaire 2 (men). | Difficulty falling asleep | | Waking up during night | | Early awakening | | Sleep medication | |
| --- | --- | --- | --- | --- | --- | --- | --- | --- |
|  | Usually | Never | Usually | Never | Usually | Never | Usually | Never |
| **All (n=5582)** | 314 (5.6)*** | 3534 (63.3) | 501 (9.0)*** | 2113 (37.9) | 652 (11.7)*** | 2103(37.7) | 199 (3.6)*** | 5171 (92.9) |
| **Age (y)** |  |  |  |  |  |  |  |  |
| 49–54 (n=232) | 9 (3.9)** | 146 (62.9) | 17 (7.3)* | 93 (40.1) | 30 (12.9) | 90(38.8) | 0 (0.0)*** | 226 (97.4) |
| 55–64 (n=1806) | 97 (5.4) | 1189 (65.8) | 157 (8.7) | 730 (40.4) | 213 (11.8) | 710(39.3) | 50 (2.8) | 1700 (94.2) |
| 65–74 (n=2024) | 116 (5.7) | 1300 (64.2) | 178 (8.8) | 762 (37.6) | 227 (11.2) | 758(37.4) | 71 (3.5) | 1896 (93.8) |
| 75–84 (n=1351) | 76 (5.6) | 799 (59.1) | 126 (9.4) | 470 (34.9) | 167 (12.4) | 480(35.6) | 64 (4.8) | 1211 (90.2) |
| 85–90 (n=167) | 16 (9.6) | 100 (59.9) | 23 (13.9) | 58 (35.1) | 15 (9.0) | 65(38.9) | 14 (8.4) | 138 (82.6) |
| **Social class** |  |  |  |  |  |  |  |  |
| Nonmanual (n=3465) | 167 (4.8)*** | 2288 (66.0) | 282 (8.1)* | 1350 (39.0) | 361 (10.4)*** | 1364(39.4) | 124 (3.6) | 3220 (93.1) |
| Manual (n=2055) | 142 (6.9) | 1212 (59.0) | 213 (10.4) | 743 (36.1) | 281 (13.7) | 721(35.1) | 73 (3.6) | 1893 (92.5) |
| **Education** |  |  |  |  |  |  |  |  |
| Lower (n=1886) | 132 (7.0)*** | 1126 (59.7) | 192 (10.2)* | 679 (36.1) | 271 (14.4)*** | 673(35.7) | 78 (4.1) | 1727 (91.9) |
| Higher (n=3694) | 181 (4.9) | 2407 (65.2) | 309 (8.4) | 1433 (38.8) | 381 (10.3) | 1430(38.7) | 120 (3.2) | 3443 (93.4) |
| **Occupation** |  |  |  |  |  |  |  |  |
| Working (n=1098) | 36 (3.3)*** | 756 (68.9) | 77 (7.0)* | 471 (42.9) | 113 (10.3) | 443(40.3) | 13 (1.2)*** | 1059 (96.4) |
| Not working (n=2580) | 148 (5.7) | 1617 (62.7) | 227 (8.8) | 967 (37.5) | 288 (11.2) | 952(36.9) | 93 (3.6) | 2392 (93.1) |
| **Marital status** | |  |  |  |  |  |  |  |
| Single (n=120) | 5 (4.2)* | 75 (63.6) | 7 (5.8)* | 64 (53.3) | 8 (6.7) | 55(45.8) | 6 (5.0)* | 108 (90.8) |
| Married (n=3149) | 151 (4.8) | 2061 (65.5) | 252 (8.0) | 1225 (39.0) | 344 (10.9) | 1184(37.6) | 84 (2.7) | 2970 (94.6) |
| Others# (n=385) | 28 (7.2) | 222 (57.5) | 42 (10.9) | 144 (37.3) | 47 (12.2) | 144(37.4) | 15 (3.9) | 351 (90.9) |
| **General health** | |  |  |  |  |  |  |  |
| Excellent (n=258) | 4 (1.5)*** | 211 (81.8) | 7 (2.7)*** | 150 (58.4) | 16 (6.2)*** | 129(50.0) | 3 (1.2)*** | 254 (98.4) |
| Very good (n=1246) | 33 (2.6) | 884 (71.0) | 68 (5.5) | 542 (43.6) | 96 (7.7) | 528(42.4) | 16 (1.3) | 1190 (95.7) |
| Good (n=1499) | 74 (4.9) | 930 (62.0) | 122 (8.1) | 555 (37.0) | 168 (11.2) | 526(35.1) | 39 (2.6) | 1420 (94.8) |
| Fair (n=582) | 57 (9.8) | 307 (52.8) | 86 (14.8) | 162 (27.9) | 101 (17.3) | 181(31.1) | 34 (5.9) | 514 (89.1) |
| Poor (n=80) | 16 (20.0) | 34 (42.5) | 22 (27.8) | 23 (29.1) | 18 (22.5) | 25(31.2) | 13 (16.5) | 62 (78.5) |
| **Preexisting diseases** | |  |  |  |  |  |  |  |
| No (n=4195) | 159 (5.3)** | 1976 (65.6) | 239 (7.9) | 1166 (38.8) | 339 (11.2) | 1135(37.7) | 92 (3.1)* | 2817 (93.7) |
| Yes## (n=946) | 96 (5.8) | 1011 (61.1) | 161 (9.7) | 618 (37.3) | 184 (11.1) | 632(38.2) | 74 (4.5) | 1513 (91.6) |

Abbreviation: y, year.

Data are presented as number (%).

**P*<.05; ***P*<.005; ****P*<.001.

For all, *is for comparisons between men and women. Comparisons were based on χ^2^ tests.

#Widowed, separated, or divorced.

##Self-reported myocardial infarction, stroke, or cancer.

| Supplementary Table 2 Sleep difficulties measured in the Health and Lifestyle Questionnaire 2 (women). | Difficulty falling asleep | | Waking up during the night | | early awakening | | Sleep medication | |
| --- | --- | --- | --- | --- | --- | --- | --- | --- |
|  | Usually | Never | Usually | Never | Usually | Never | Usually | Never |
| **All (n=7315)** | 774 (10.6) | 2889 (39.6) | 1076 (14.7) | 1854 (25.4) | 1076 (14.7) | 2270 (31.0) | 414 (5.7) | 6414 (87.7) |
| **Age (y)** |  |  |  |  |  |  |  |  |
| 49–54 (n=373) | 42 (11.3)*** | 183 (49.1) | 69 (18.5) | 101 (27.1) | 69 (18.5)*** | 130 (34.8) | 21 (5.6)*** | 332 (89.0) |
| 55–64 (n=2669) | 283 (10.6) | 1097 (41.1) | 392 (14.7) | 708 (26.5) | 363 (13.6) | 902 (33.7) | 128 (4.8) | 2386 (89.3) |
| 65–74 (n=2511) | 236 (9.4) | 982 (39.1) | 349 (13.9) | 631 (25.2) | 347 (13.8) | 750 (29.8) | 139 (5.5) | 2224 (88.6) |
| 75–84 (n=1547) | 179 (11.6) | 549 (35.5) | 231 (14.9) | 359 (23.1) | 261 (16.8) | 425 (27.4) | 108 (6.9) | 1304 (83.8) |
| 85–90 (n=200) | 34 (17.0) | 78 (39.0) | 35 (17.1) | 55 (26.8) | 36 (18.1) | 63 (31.7) | 18 (8.9) | 168 (82.8) |
| **Social class** |  |  |  |  |  |  |  |  |
| Nonmanual (n=4591) | 428 (9.3)*** | 1978 (43.1) | 623 (13.6)*** | 1235 (26.9) | 607 (13.2)*** | 1511 (32.9) | 265 (5.8) | 4030 (87.6) |
| Manual (n=2623) | 333 (12.7) | 874 (33.4) | 438 (16.7) | 598 (22.8) | 451 (17.2) | 730 (27.8) | 143 (5.5) | 2297 (87.7) |
| **Education** |  |  |  |  |  |  |  |  |
| Lower (n=3460) | 440 (12.8)*** | 1152 (33.4) | 577 (16.7)*** | 763 (22.1) | 585 (16.9)*** | 918 (26.5) | 220 (6.3)** | 2989 (86.3) |
| Higher (3851) | 334 (8.7) | 1736 (45.1) | 499 (12.9) | 1091 (28.3) | 491 (12.7) | 1352 (35.1) | 194 (5.0) | 3425 (88.9) |
| **Occupation** |  |  |  |  |  |  |  |  |
| Working (n=1083) | 94 (8.7)** | 494 (45.6) | 161 (14.9)* | 307 (28.3) | 138 (12.7)* | 380 (35.1) | 35 (3.2)*** | 993 (91.8) |
| Not working (n=3716) | 373 (10.0) | 1476 (39.7) | 506 (13.6) | 917 (24.7) | 508 (13.7) | 1126 (30.3) | 211 (5.7) | 3254 (87.6) |
| **Marital status** | |  |  |  |  |  |  |  |
| Single (n=203) | 22 (10.8)** | 89 (43.8) | 25 (12.3)*** | 72 (35.5) | 23 (11.3)*** | 81 (39.9) | 12 (5.9)* | 179 (88.6) |
| Married (n=3294) | 291 (8.8) | 1387 (42.1) | 426 (13.0) | 824 (25.1) | 408 (12.4) | 1042 (31.6) | 147 (4.5) | 2943 (89.5) |
| Others (n=1285) | 153 (11.9) | 487 (37.9) | 211 (16.3) | 319 (24.7) | 210 (16.3) | 373 (29.0) | 84 (6.5) | 1115 (86.4) |
| **General health** | |  |  |  |  |  |  |  |
| Excellent (n=347) | 14 (4.0)*** | 211 (60.8) | 29 (8.4)*** | 132 (28.0) | 28 (8.1)*** | 147 (42.4) | 10 (2.9)*** | 328 (95.1) |
| Very good (n=1663) | 103 (6.2) | 762 (45.8) | 166 (10.0) | 493 (29.6) | 167 (10.0) | 590 (35.5) | 40 (2.4) | 1541 (92.7) |
| Good (n=1966) | 204 (10.4) | 732 (37.3) | 282 (14.3) | 457 (23.2) | 260 (13.2) | 582 (29.6) | 114 (5.8) | 1721 (87.5) |
| Fair (n=712) | 122 (17.2) | 232 (32.7) | 159 (22.5) | 120 (17.0) | 161 (22.6) | 160 (22.5) | 64 (9.0) | 577 (81.0) |
| Poor (n=98) | 23 (23.5) | 27 (27.5) | 29 (29.3) | 18 (18.2) | 25 (25.5) | 18 (18.4) | 16 (16.2) | 70 (70.7) |
| **Preexisting diseases** | |  |  |  |  |  |  |  |
| No (n=4065) | 384 (9.4)** | 1704 (41.9) | 548 (13.5)* | 1098 (27.0) | 542 (13.3)*** | 1362 (33.5) | 192 (4.7)* | 3616 (88.9) |
| Yes (n=2184) | 240 (11.0) | 812 (37.2) | 331 (15.2) | 529 (24.2) | 335 (15.3) | 614 (28.1) | 143 (6.5) | 1908 (87.4) |

Abbreviation: y, year.

Data are presented as number (%).

**P*<.05; ***P*<.005; ****P*<.001.

For all, *is for comparisons between men and women. Comparisons were based on χ^2^ tests.

#Widowed, separated, or divorced.

##Self-reported myocardial infarction, stroke, or cancer.

| Supplementary Table 3 Comparison of sleep time of European Prospective Investigation of Cancer -Norfolk participants with other populations. | Country | Year | Age | Gender | Bedtime |  | Rise time |  | Time in bed | | Sleep duration | |
| --- | --- | --- | --- | --- | --- | --- | --- | --- | --- | --- | --- | --- |
|  | (N) |  |  |  | Weekdays | Weekends | Weekdays | Weekends | Weekdays | Weekends | Weekdays | Weekends |
| EPIC-Norfolk | UK | 2006-2007 | 46-90 | Men | 22:46(0.82) | 22:57(0.85) | 7:13(0.91) | 7:40(0.88) | 8.46(0.97) | 8.71(0.97) | 6.99 (1.11) | |
|  | (8480) |  |  | Women | 22:37(0.77) | 22:49(0.80) | 7:20(0.82) | 7:46(0.84) | 8.72(0.92) | 8.96(0.92) | 6.80 (1.19) | |
| Hordaland Health study [1] | Norway | 1997-1999 | 40-45 | Men | 23:13(0.46) | 00:02(0.54) | 6:23(0.51) | 8:22(1.06) | 7.10(0.54) | 8.20(1.03) | 6.52(0.55) | 8.02(1.06) |
|  | (8860) |  |  | Women | 23:05(0.41) | 00:01(0.51) | 6:37(0.48) | 8:39(1.02) | 7.31(0.54) | 8.37(1.03) | 7.11(0.57) | 8.18(1.05) |
| Ohayon 2004 [2] | 7 European countries  (8089) | | 55-101 | Men | 22:54 |  | 6:46 |  | 7.87 |  | 7.00 (1.40) | |
|  |  |  |  | Women | 22:48 |  | 6:54 |  | 8.10 |  | 6.88 (1.47) | |

Abbreviation: EPIC, European Prospective Investigation of Cancer.

Data are presented as number (%).

**P*<.05; ***P*<.005; ****P*<.001.

For total, *is for comparisons between men and women.

#Widowed, separated, or divorced.

##Self-reported myocardial infarction, stroke, or cancer.

Bedtime and rise time: results are presented as mean (hour: minute) (standard deviation [hour]).

Time in bed and sleep duration: results are presented as mean (hour) (standard deviation [hour]).

[1] Ursin R, Bjorvatn B, Holsten F. Sleep duration, subjective sleep need, and sleep habits of 40- to 45-year-olds in the Hordaland Health Study. Sleep 2005;28:1260-9.

[2] Ohayon MM. Interactions between sleep normative data and sociocultural characteristics in the elderly. J Psychosom Res 2004;56:479-86.
